# Confronting Vaccine Hesitancy in India: A Vital Call for Unified Action

**DOI:** 10.7759/cureus.73459

**Published:** 2024-11-11

**Authors:** Sheuli Paul, Shradha Salunkhe, Shailaja V Mane, Mridu Bahal

**Affiliations:** 1 Paediatrics, Dr. D. Y. Patil Medical College, Hospital & Research Centre, Dr. D. Y. Patil Vidyapeeth (Deemed to be University), Pune, IND

**Keywords:** booster doses, community engagement, cultural beliefs, digital literacy, immunization coverage, infectious diseases, misinformation, public health, vaccine, vaccine hesitancy

## Abstract

In India, vaccine hesitancy is one of the major hurdles to attaining widespread immunity against communicable diseases. Vaccines play a significant role, as was evident during the COVID-19 pandemic. However, factors such as misinformation, cultural beliefs together with distrust in healthcare providers are some of the barriers encountered during mass vaccination. Education and communication strategies based on evidence are the need of the hour to address this issue. Enhancing vaccination rates requires a multi-pronged strategy of reclaiming control: through digital literacy, community engagement, and clear communication by healthcare personnel as well as the government. The recent resurgence of vaccine-preventable diseases in the country underscores the need for booster doses to combat waning immunity. Coordinated efforts between all the key players are required to confront vaccine hesitancy and to ensure that vaccines provide better health for all.

## Editorial

As evident from the recent COVID-19 pandemic, vaccines played a key role in protecting the lives and health of citizens. In 2019, the World Health Organization declared vaccine hesitancy as one of the top 10 threats to global health, recognizing that hesitance or rejection to vaccinate undermines progress toward ensuring all children receive vital immunizations [1]. In India, vaccine hesitancy remains a major roadblock to achieving herd immunity for many infectious diseases. Such hesitancy - fuelled largely by misinformation, collusion of cultural beliefs, and national distrust in the healthcare system itself - has begun to impede the nation's continued fight against infectious diseases. Comprehensive, evidence-based education is crucial to addressing vaccine hesitancy. The latest National Family Health Survey (NFHS-5, 2019-21) reveals that India's full immunization coverage stands at just 76.4%, meaning that a staggering one in four children is being denied access to essential vaccines [2]. Each year, 500,000 children die in India from vaccine-preventable diseases, and another 8.9 million remain at risk due to partial or no immunization [3]. This stark reality underscores the urgent need for decisive action to improve vaccination rates and safeguard the health of our nation's children. Curbing vaccine hesitancy in India is a complex challenge with multiple facets and can only be solved by public education and social engagement along with open communication from the government as well as healthcare providers.

The phenomenon of vaccine hesitancy in India is not new, and it has affected several vaccines over time, including polio and measles [4]. The COVID-19 pandemic has demonstrated the risk that complacency creates, born from decades of vaccine hesitancy and low levels of investment in new vaccines. The COVID-19 pandemic, along with the challenges and vaccination campaigns it precipitated, placed significant strain on healthcare systems in 2020 and 2021, leading to pronounced setbacks. Data from 2023 indicate that performance levels have yet to recover to their pre-pandemic 2019 benchmarks [5]. WHO/UNICEF Estimates of National Immunization Coverage (WUENIC) stated that the number of zero-dose children in India rose from 1.3 million during the pre-pandemic times to 2.7 million in 2021. In 2023, at 1.6 million, India had the second-highest number of zero-dose (ZD) children in the world [6]. A recent study by Johns Hopkins University shows that vaccine hesitancy is significant in India, driven mainly by fears of side effects or adverse events and doubts about whether the vaccines are effective with a lot of hesitancy attributed to misinformation [7]. The recent resurgence of vaccine-preventable diseases such as measles and diphtheria highlights the detrimental consequences of suboptimal vaccination rates [8]. These outbreaks emphasize the critical importance of maintaining adequate immunity through routine immunization and booster doses to safeguard public health [9].

The leading cause of vaccine hesitancy is misinformation [10,11]. Misinformation is already rampant in the digital age and typically spreads more quickly via social media than does accurate information. Fear of potential side effects, rumors about the contents of vaccines and conspiracy theories, or misinformation on vaccine efficacy also drive fear. In a more recent context, the COVID-19 pandemic offered critical lessons in addressing vaccine controversies and hesitancy. The rapid development and deployment of COVID-19 vaccines led to both groundbreaking achievements in public health and considerable public debate. Multiple studies explored these challenges, from initial skepticism about the vaccines' speed of development to concerns over safety and effectiveness [12]. The responses from health authorities, which included extensive public health campaigns, transparent sharing of vaccine trial data, and close monitoring of adverse effects, demonstrated effective strategies for tackling vaccine hesitancy. In such situations, it is necessary to rely on a powerful digital literacy campaign that informs people about ways to recognize the authenticity of information and dispel most myths. Similarly, cultural and religious beliefs are significant determinants of acceptability towards the usage of vaccines. Traditional beliefs and practices pre-date available medical interventions in some communities. Working with local political leaders, religious figures and community influencers can be a first step to connect these two worlds. These figures are trusted and can stand as spokespersons for the vaccines, delivering culturally appropriate messages that speak to their communities [13].

In addition, the longstanding history of distrust for the healthcare system only increases hesitancy in making an ill-advised decision when it comes to vaccinating. Reports of medical malpractice, barriers to health access, and perception of negligence by authorities have made a lot more Indians wary of state-backed healthcare schemes. For people to trust, health officials need to be communicating consistently, transparently, and with empathy. It is very important that the government be in touch with communities, really listen to them, and resolve their concerns. That means acknowledging where we have fallen short in the past and taking steps to ensure that health care is provided equitably. Education has been empirically shown to be a decisive factor in combating vaccine hesitancy. Raising awareness through comprehensive, evidence-based educational campaigns can greatly contribute to overcoming this challenge. This includes a holistic and inclusive public health campaign that speaks to all demographics. Informative sessions and workshops at schools, workplaces, or community centers can further broaden the appeal of such initiatives through the use of language and regional dialect-based translations.

In an era where misinformation spreads rapidly, responsible journalism plays a crucial role in promoting public health and fighting vaccine hesitancy. Sensationalist headlines or one-sided narratives often mislead the public, potentially discouraging vaccination or fostering unnecessary alarm. Instead, journalists should prioritize balanced reporting that presents both the benefits and potential risks of vaccines in an objective manner. For instance, when covering side effects or adverse events, it is essential to contextualize them with data on their rarity and the overall safety profile of vaccines, based on scientific evidence. This empowers audiences to make informed decisions rather than be swayed by fear. Providing a "full counterbalance" in vaccine reporting means including perspectives from credible sources, such as health professionals, scientists, and public health experts, alongside addressing public concerns. When information is presented in a fact-based, unbiased manner, it builds trust with readers. Furthermore, by debunking myths and clarifying misinformation within these articles, journalists contribute to a more informed public discourse around vaccination [14]. Goodwill among the public can be cultivated using other modalities as well, and it is this multi-pronged approach that India needs to foster in order to address vaccine hesitancy. With an approach that is focused on improving digital literacy, bolstering community leaders, and establishing trust in the healthcare system by using inclusive communication strategies/messaging - it becomes possible to break these barriers down. Therefore, it is essential to have a concerted global initiative to ensure that vaccines continue to deliver on their long-standing promise of preserving public health and saving lives. In the history of medicine, few interventions have had as great an impact on the morbidity and mortality of populations as vaccination. Hence, vaccines are arguably one of the best tools available in contemporary medicine. We can build a healthier tomorrow for everyone by uniting to tackle vaccine hesitancy and bolster immunization campaigns.
